# Supplementary material for: BAT26 Only Microsatellite Instability with High Tumor Mutation Burden—A Rare Entity Associated with PTEN Protein Loss and High PD-L1 Expression
Source: Int J Mol Sci. 2022 Sep 14;23(18):10730. doi: 10.3390/ijms231810730 (PMC9504117; doi:10.3390/ijms231810730)
Supplement: Supplementary file 1 [file ijms-23-10730-s001.zip › ijms-1894791-supplementary.pdf]

Supplementary Table S1. Somatic mutations of cases with BAT26-only microsatellite instability with high tumor mutation burden

	Tier	Gene	RefSeq	AA	Nucleotide	Total	Variant	VAF	Drug
Case 01	IIC	KRAS	NM_033360.3	p.Gly12Ala	c.35G>C	448	85	19.0%	Panitumumab, Cetuximab
	IIC	KRAS	NM_033360.3	p.Gly12Ala	c.35G>C	448	85	19.0%	Cobimetinib
	IID	KRAS	NM_033360.3	p.Gly12Ala	c.35G>C	448	85	19.0%	Cobimetinib, Binimetinib, Trametinib
	III	RAD51	NM_133487.3	p.Arg151Gln	c.452G>A	516	246	47.7%	
	III	AKT1	NM_001014431.1	p.Trp80Arg	c.237T>C	1465	39	2.7%	
	III	IRS2	NM_003749.2	p.Ala624Thr	c.1870G>A	1493	54	3.6%	
	III	IRS2	NM_003749.2	p.Ala1053Thr	c.3157G>A	770	34	4.4%	
	III	EED	NM_001308007.1	p.Phe289Leu	c.867T>A	262	15	5.7%	
	III	MEN1	NM_000244.3	p.Glu392Lys	c.1174G>A	1332	44	3.3%	
	III	ARID5B	NM_032199.2	p.Glu612del	c.1836_1838del	1839	1327	0.7216	
	III	PTEN	NM_001304717.2	p.Arg303Pro	c.908G>C	385	171	0.4442	
	III	ANKRD11	NM_013275.5	p.Ala2030Gly	c.6089C>G	992	478	0.4819	
	III	ANKRD26	NM_014915.2	p.Asp313Asn	c.937G>A	449	183	0.4076	
	III	ANKRD26	NM_014915.2	p.Lys1234AsnfsTer19	c.3702del	588	34	5.8%	
	III	SOX17	NM_022454.3	p.Ser105del	c.314_316del	802	38	4.7%	
	III	NCOR1	NM_006311.3	p.Arg1113Gln	c.3338G>A	350	197	56.3%	
	III	SETBP1	NM_015559.2	p.Thr510Ala	c.1528A>G	1060	54	5.1%	
	III	AXIN2	NM_004655.3	p.Arg834Gln	c.2501G>A	1181	66	5.6%	
	III	SMAD2	NM_005901.5	p.Cys149Arg	c.445T>C	365	18	4.9%	
	III	DOT1L	NM_032482.2	p.His1461Tyr	c.4381C>T	666	18	2.7%	
	III	MAP2K2	NM_030662.3	p.Ala315Thr	c.943G>A	987	52	5.3%	
	III	DNMT1	NM_001130823.2	p.Gly876Arg	c.2626G>A	1156	572	49.5%	
	III	CALR	NM_004343.3	p.Thr350Ile	c.1049C>T	1493	55	3.7%	
	III	PIK3R2	NM_005027.3	p.Gln117Lys	c.349C>A	988	41	4.2%	
	III	EWSR1	NM_013986.3	p.Gly602Cys	c.1804G>T	1966	363	18.5%	
	III	EP300	NM_001429.3	p.Gly322Ala	c.965G>C	1286	636	49.5%	
	III	EP300	NM_001429.3	p.Val456Ile	c.1366G>A	717	334	46.6%	
	III	EP300	NM_001429.3	p.Met2161Val	c.6481A>G	1494	772	51.7%	
	III	ARAF	NM_001256196.1	p.Arg282Cys	c.844C>T	1751	26	1.5%	
	III	MAP3K14	NM_003954.4	p.Asp852Asn	c.2554G>A	1586	760	47.9%	
	III	SMO	NM_005631.4	p.Ser699Asn	c.2096G>A	1569	66	4.2%	
	III	MYB	NM_001130173.1	p.Ala252Ser	c.754G>T	783	33	4.2%	
	III	ETV1	NM_004956.4	p.Gly321Ter	c.961G>T	549	18	3.3%	

	III	PIK3CD	NM_005026.3	p.Ala414Val	c.1241C>T	1611	20	1.2%	
	III	ARID1A	NM_006015.4	p.Gln1519ProfsTer13	c.4555dup	1526	276	18.1%	
	III	ARID1A	NM_006015.4	p.Asp1850ThrfsTer33	c.5548del	1499	19	1.3%	
	III	FUBP1	NM_001303433.1	p.Leu364del	c.1091_1093del	333	18	5.4%	
	III	SPTA1	NM_003126.2	p.Arg28Cys	c.82C>T	678	15	2.2%	
	III	PIK3C2B	NM_002646.3	p.Asp374MetfsTer5	c.1120del	1361	28	2.1%	
	III	IKBKE	NM_014002.3	p.Cys545Phe	c.1634G>T	1401	534	38.1%	
	III	PARP1	NM_001618.3	p.Ala237ProfsTer2	c.708del	784	28	3.6%	
	III	HIST3H3	NM_003493.2	p.Phe68Ter	c.203_204delinsAA	2042	1571	76.9%	
	III	ASXL2	NM_018263.4	p.Gly1134Ser	c.3400G>A	1093	470	43.0%	
	III	RANBP2	NM_006267.4	p.Thr2398Ser	c.7192A>T	389	168	43.2%	
	III	ERBB4	NM_005235.2	p.Glu314Lys	c.940G>A	420	17	4.0%	
	III	PAX3	NM_181459.3	p.Lys183del	c.547_549del	2321	108	4.7%	
	III	MAGI2	NM_012301.3	p.Gln635Leu	c.1904A>T	1198	16	1.3%	
	III	PDCD1	NM_005018.2	p.Thr36HisfsTer70	c.105dup	1145	59	5.2%	
	III	SETD2	NM_014159.6	p.Arg540Ter	c.1618C>T	496	33	6.7%	
	III	ETV5	NM_004454.2	p.Ala192Glu	c.575C>A	1418	93	6.6%	
	III	FBXW7	NM_018315.4	p.Arg59ThrfsTer37	c.175_179del	538	122	22.7%	
	III	FBXW7	NM_033632.3	p.Val8Met	c.22G>A	419	17	4.1%	
	III	MAP3K1	NM_005921.1	p.Pro138Ser	c.412C>T	156	10	6.4%	
	III	PIK3R1	NM_181523.2	p.Thr576del	c.1727_1729del	375	33	8.8%	
	III	APC	NM_000038.5	p.Arg2525Cys	c.7573C>T	424	87	20.5%	
	III	NSD1	NM_022455.4	p.Pro2225GlnfsTer69	c.6674del	1398	59	4.2%	
	III	HIST1H1C	NM_005319.3	p.Ala189Val	c.566C>T	1168	463	39.6%	
	III	HIST1H3G	NM_003534.2	p.Arg43His	c.128G>A	1833	56	3.1%	
	III	PRDM1	NM_001198.3	p.Arg302Gln	c.905G>A	1464	55	3.8%	
	III	ROS1	NM_002944.2	p.Ser1109Leu	c.3326C>T	509	284	55.8%	
	III	KDM5C	NM_004187.3	p.Leu938Pro	c.2813T>C	1776	20	1.1%	
	III	SETD2	NM_014159.6	p.Gln2127Arg	c.6380A>G	992	15	1.5%	
	III	AR	NM_000044.3	p.Leu57del	c.170_172del	437	44	10.1%	
	Alterations of HR related genes variant	PTEN	NM_001304717.2	p.Arg303Pro	c.908G>C	385	171	44.4%	
		ARID1A	NM_006015.4	p.Gln1519ProfsTer13	c.4555dup	1526	276	18.1%	
		ARID1A	NM_006015.4	p.Asp1850ThrfsTer33	c.5548del	1499	19	1.3%	
Case 02	IA	KRAS	NM_033360.3	p.Ala146Thr	c.436G>A	259	121	46.7%	Panitumumab, Cetuximab
	IIC	KRAS	NM_033360.3	p.Ala146Thr	c.436G>A	259	121	46.7%	Cobimetinib
	IID	KRAS	NM_033360.3	p.Ala146Thr	c.436G>A	259	121	46.7%	Cobimetinib, Binimetinib, Trametinib
	III	TP53	NM_000546.5	p.Arg282Trp	c.844C>T	394	144	36.5%	
	III	FLT1	NM_002019.4	p.Glu144Lys	c.430G>A	340	174	51.2%	

	III	ERBB3	NM_001982.3	p.Arg1127His	c.3380G>A	847	304	35.9%	
	III	HRAS	NM_001130442.2	p.Ala122Val	c.365C>T	1066	591	55.4%	
	III	ABL1	NM_007313.2	p.Ser901del	c.2700_2702del	792	534	67.4%	
	III	CDKN2A	NM_001195132.1	p.His158Asn	c.472C>A	374	153	40.9%	
	III	KAT6A	NM_001099412.1	p.Gly361Val	c.1082G>T	382	260	68.1%	
	III	APC	NM_000038.5	p.Glu1353Ter	c.4057G>T	278	96	34.5%	
	III	HIST1H1C	NM_005319.3	p.Ala8Thr	c.22G>A	631	54	8.6%	
	III	AXIN2	NM_004655.3	p.Ser738Phe	c.2213C>T	528	278	52.7%	
	III	MSH2	NM_000251.2	p.Gln629Arg	c.1886A>G	262	141	53.8%	
	III	MSH2	NM_000251.2	p.Gln419Lys	c.1255C>A	254	144	56.7%	
	III	EPCAM	NM_002354.2	p.Arg153Thr	c.458G>C	261	93	35.6%	
	III	MUTYH	NM_001128425.1	p.Pro18Leu	c.53C>T	365	159	43.6%	
	III	MUTYH	NM_001128425.1	p.Gly25Asp	c.74G>A	387	167	43.2%	
	III	PRDM1	NM_001198.3	p.Arg651Ter	c.1951C>T	460	71	15.4%	
	III	STK11	NM_000455.4	p.Arg426Trp	c.1276C>T	696	160	23.0%	

Supplementary Table S2. Microsatellite loci in BAT26 only- microsatellite instability cases by TruSight Oncology 500 NGS test

Case	Gene	Chromosome Number	Start	Repeat Unit	Assessed	Distance	P-Value
case 01	PAX8	chr2	113982124	A	TRUE	0.1224098	3.84731E-45
	PAX8	chr2	113991269	A	TRUE	0.1367512	3.5039E-128
	FOXP1	chr3	71007462	T	TRUE	0.1426004	1.5041E-152
	E2F3	chr6	20443198	A	TRUE	0.1177951	1.30063E-23
	ROS1	chr6	117642992	T	TRUE	0.12262	5.37679E-18
	MYB	chr6	135526367	T	TRUE	0.1273972	5.67029E-46
	MYB	chr6	135528875	T	TRUE	0.1123496	2.82871E-17
	MYB	chr6	135529684	T	TRUE	0.1082472	4.12195E-19
	ETV1	chr7	13988790	A	TRUE	0.1904444	3.45822E-15
	CASC11	chr8	128744519	T	TRUE	0.1214149	2.67922E-17
	MYC	chr8	128751902	T	TRUE	0.1107615	2.0214E-06
	FLI1	chr11	128681586	T	TRUE	0.1526945	5.69015E-24
	EWSR1	chr22	29682881	T	TRUE	0.1157463	2.75202E-39
	EWSR1	chr22	29696468	T	TRUE	0.1157659	5.83761E-69
case 02	MYB	chr6	135538507	A	TRUE	0.1114455	3.87031E-05
	FGFR2	chr10	123240429	A	TRUE	0.1352151	3.8244E-140
	FLI1	chr11	128681586	T	TRUE	0.129524	7.62105E-18
	LAMP1	chr13	113964898	T	TRUE	0.1299736	2.47455E-39

NGS, Next generation sequencing

Supplementary Table S3. Summary of studies showing MSI-L in published literatures

No. of Study	Authors	Years	Cancer Type	Method	Microsatellite Markers	MSI Cutoff Value	MSI-L cases (%)	
1	Our study	2021	various solid tumor	PCR	5 quasimonomorphic marker in PCR and TSO500	at least one and 10> and <20% unstable marker in TSO500	124/6,476 (1.9%) or 96/4,730 (2.0%) in colorectal cancer	BAT26 only unstable TMB-high
2	TCGA [1]	2014	gastric	WES and WGS	Seven polymorphic markers (BAT25, BAT26, BAT40, TGFBRII, D2S123, D5S346, and D17S250)	one or two altered markers (<40%)		
3	Kim et al [2]	2013	colorectal and endometrial	WES and WGS		median of 5 and 2 MSI events per CRC and EC genomes	23/147 (15.6%) in CRC, 11/130 (0.8%) in EC	grouped MSI-L with MSS
4	TCGA [3]	2013	endometrial	WES and WGS	Seven polymorphic markers (BAT25, BAT26, BAT40, TGFBRII, D2S123, D5S346, and D17S250)	one or two altered markers (<30%)		
5	TCGA [4]	2012	colorectal	WES and WGS	Seven polymorphic markers (BAT25, BAT26, BAT40, TGFBRII, D2S123, D5S346, and D17S250) and MSI Analysis System (Promega); (BAT25, BAT26, NR21, NR24, and NR27)	one or two altered markers (<30%)		
6	Chapelle et al [5]	2010	colorectal	PCR	5 Bethesda Panel (BAT25, BAT26, D2S123, D5S346 and D17S250)	<40%; at least one	107/1,566 (6.8%)	most or all MSI-L tumors have no MMR defect
7	Kim et al [6]	2009	colorectal	PCR	5 Bethesda Panel	at least one	30/657 (4.6%)	less frequent lymph node metastasis and advanced tumor stage than MSS CRCs
8	Hampel et al [7]	2008	colorectal	PCR	5 Bethesda Panel	<40%; at least one	34/500 (6.8%)	
9	Hampel et al [8]	2005	colorectal	PCR	six polymorphic markers (BAT25, BAT26, D2S123, D5S346, and D18S69 or D17S250	<40%; at least one	73/1,066 (6.8%)	

10	Kohonen-Corish et al [9]	2005	colorectal	PCR	six markers BAT25, BAT26, D2S123, D5S346, D17S250, and MYCL1	at least one	51/183 (27.9%)	poorer survival, Loss of MGMT protein expression
11	Wright et al [10]	2005	colorectal	PCR	11 microsatellites (BAT25,7 BAT26,7 BAT40,7 D2S123,32 D5S346,33 D10S197,32 D17S250,34 D18S58,35 D18S69,32 c-myb,36 and L-myc37)	<40%; at least one	33/255 (12.9%)	Sporadic MSI-L and MSS CRCs have comparable clinicopathological features.
12	Pawlik et al [11]	2004	Review	PCR	various mono-, di-nucleotide repeat	<30-40%;		MSI-L tumors differ quantitatively from MSS tumors but do not differ qualitatively
13	Halford et al [12]	2003	colorectal, endometrial, breast and ovarian	PCR	six polymorphic markers (D2S123, D5S346, D10S197, D13S175, D18S69, D20S100, D15S659, and MYCL)	<30%	30/87 (35%) colorectal cancers, 3/94 (3%) breast carcinomas, 16/50 (32%) endometrial cancers, 9/34 (26%) ovarian cancers	
14	Laiho et al [13]	2002	colorectal	PCR	377 markers	11 markers	71/90 (79%) in BAT26 stable	No difference between MSI-L and MSS
15	Tomlinson et al [14]	2002	colorectal	PCR	mono-, di-nucleotide repeat	review	3-35%	frequency of KRAS mutations
16	Halford [15]	2002	colorectal	PCR	44 microsatellites	<30%;at least one	68% in the non-MSI-H group showed MSI-L	MSI-L could be used to trace the evolution of almost every cancer of any site

17	Whitehall et al [16]	2001	colorectal	PCR	5 Bethesda Panel	1 unstable, or MYCL and one dinucleotide unstable	44/90 (48.9%)	No difference between KRAS mutation in MSI-L and MSS (p>0.2), but MGMT methylation more frequent in MSI-L than MSS (p>0.005)
18	Alexander et al [17]	2001	colorectal	PCR	8 dinucleotide repeats (D18S55, D18S58, D18S61, D18S64, D18S69, D17S1176, D17S520, TP53) and 2 mononucleotide repeats (TGF-β1-RII, BAT26)	Any 1 unstable (not BAT26 or TGF-B1-RII)	520	Grouped MSI-L and MSS together
19	Gonzalez-Garcia et al [18]	2000	colorectal	PCR	six microsatellite markers (BAT26, SIT2, D21S415, D21S1235, D12S95, D4S2948) and 22 CA repeats	Any 1 unstable	33/415 (8%)	Chose the primers as ones most commonly positive in the larger screen of 26 markers. No statistical evidence for separate MSI-L group in terms of sex, age, stage, site, differentiation p53 mutations, and KRAS
20	Gryfe et al [19]	2000	colorectal	PCR	BAT-25, BAT-26, D5S346, D2S123, D17S250, BAT-40, TGF-β RII, D18S58, D18S69, and D17S787	1 to 3 unstable of 10 loci	20/607 (3%)	Clinicopathological features similar in MSI-L and MSS
21	Jass et al [20]	1999	colorectal	PCR	six polymorphic markers (BAT26, BAT40, AT3, D2S123, F138, MYCL)	<40%; at least one	38/114 (33.3%)	MSI-L colorectal cancers are distinct from both MSI-H and MSS cancers

22	Mirabelli-Primdahl et al [21]	1999	colorectal and endometrial	PCR	5 Bethesda Panel and If one locus had MSI, up to five additional loci were tested (BAT40, BATRII, D18S58, D18S69, and D17S787) in CRCs and list (BAT-26, BAT-40, BAT RII, D2S123, D3S1611, D5S346, D17S787, D18S59, and D18S69) in Ecs	1 or 1 marker unstable in first group and 1–3 markers unstable overall	27/80 (33.8%)		No beta-catenin mutation in MSS or MSI-L cancers
23	Dietmaier et al [22]	1997	colorectal	PCR	31 microsatellite markers	<10% unstable	12/58 (20.7%)		First 5 markers were classified as MSI if 2+ loci +vs, uncertain if 1 locus + vs. & MSS if 0 +vs.

MSI-H, microsatellite instability-high; MSI-L, microsatellite instability-low; MSS, microsatellite stability

References

1. Network, C.G.A.R. Comprehensive molecular characterization of gastric adenocarcinoma. *Nature* **2014**, *513*, 202.
2. Kim, T.-M.; Laird, P.W.; Park, P.J. The landscape of microsatellite instability in colorectal and endometrial cancer genomes. *Cell* **2013**, *155*, 858-868.
3. Levine, D.A. Integrated genomic characterization of endometrial carcinoma. *Nature* **2013**, *497*, 67-73.
4. Network, C.G.A. Comprehensive molecular characterization of human colon and rectal cancer. *Nature* **2012**, *487*, 330.
5. de la Chapelle, A.; Hampel, H. Clinical relevance of microsatellite instability in colorectal cancer. *J Clin Oncol* **2010**, *28*, 3380-3387.
6. Kim, J.H.; Shin, S.H.; Kwon, H.J.; Cho, N.Y.; Kang, G.H. Prognostic implications of CpG island hypermethylator phenotype in colorectal cancers. *Virchows Archiv* **2009**, *455*, 485-494.
7. Hampel, H.; Frankel, W.L.; Martin, E.; Arnold, M.; Khanduja, K.; Kuebler, P.; Clendenning, M.; Sotamaa, K.; Prior, T.; Westman, J.A.; et al. Feasibility of Screening for Lynch Syndrome Among Patients With Colorectal Cancer. *J Clin Oncol* **2008**, *26*, 5783-5788.
8. Hampel, H.; Frankel, W.L.; Martin, E.; Arnold, M.; Khanduja, K.; Kuebler, P.; Nakagawa, H.; Sotamaa, K.; Prior, T.W.; Westman, J.; et al. Screening for the Lynch syndrome (hereditary nonpolyposis colorectal cancer). *N Engl J Med* **2005**, *352*, 1851-1860.
9. Kohonen-Corish, M.R.J.; Daniel, J.J.; Chan, C.; Lin, B.P.C.; Kwun, S.Y.; Dent, O.F.; Dhillon, V.S.; Trent, R.J.A.; Chapuis, P.H.; Bokey, E.L. Low microsatellite instability is associated with poor prognosis in stage C colon cancer. *J Clin Oncol* **2005**, *23*, 2318-2324.
10. Wright, C.M.; Dent, O.F.; Newland, R.C.; Barker, M.; Chapuis, P.H.; Bokey, E.L.; Young, J.P.; Leggett, B.A.; Jass, J.R.; Macdonald, G.A. Low level microsatellite instability may be associated with reduced cancer specific survival in sporadic stage C colorectal carcinoma. *Gut* **2005**, *54*, 103-108.
11. Pawlik, T.M.; Raut, C.P.; Rodriguez-Bigas, M.A. Colorectal carcinogenesis: MSI-H versus MSI-L. *Dis Markers* **2004**, *20*, 199-206.
12. Halford, S.E.; Sawyer, E.J.; Lambros, M.B.; Gorman, P.; Macdonald, N.D.; Talbot, I.C.; Foulkes, W.D.; Gillett, C.E.; Barnes, D.M.; Akslen, L.A.; et al. MSI-low, a real phenomenon which varies in frequency among cancer types. *J Pathol* **2003**, *201*, 389-394.
13. Laiho, P.; Launonen, V.; Lahermo, P.; Esteller, M.; Guo, M.; Herman, J.G.; Mecklin, J.P.; Järvinen, H.; Sistonen, P.; Kim, K.M.; et al. Low-level microsatellite instability in most colorectal carcinomas. *Cancer Res* **2002**, *62*, 1166-1170.
14. Tomlinson, I.; Halford, S.; Aaltonen, L.; Hawkins, N.; Ward, R. Does MSI-low exist? *J Pathol* **2002**, *197*, 6-13.
15. Halford, S.; Sasieni, P.; Rowan, A.; Wasan, H.; Bodmer, W.; Talbot, I.; Hawkins, N.; Ward, R.; Tomlinson, I. Low-level microsatellite instability occurs in most colorectal cancers and is a nonrandomly distributed quantitative trait. *Cancer Res* **2002**, *62*, 53-57.
16. Whitehall, V.L.; Walsh, M.D.; Young, J.; Leggett, B.A.; Jass, J.R. Methylation of O-6-methylguanine DNA methyltransferase characterizes a subset of colorectal cancer with low-level DNA microsatellite instability. *Cancer Res* **2001**, *61*, 827-830.
17. Alexander, J.; Watanabe, T.; Wu, T.T.; Rashid, A.; Li, S.; Hamilton, S.R. Histopathological identification of colon cancer with microsatellite instability. *Am J Pathol* **2001**, *158*, 527-535.
18. Gonzalez-Garcia, I.; Moreno, V.; Navarro, M.; Marti-Rague, J.; Marcuello, E.; Benasco, C.; Campos, O.; Capella, G.; Peinado, M.A. Standardized approach for microsatellite instability detection in colorectal carcinomas. *J Natl Cancer Inst* **2000**, *92*, 544-549.
19. Gryfe, R.; Gallinger, S. Microsatellite instability, mismatch repair deficiency, and colorectal cancer. *Surgery* **2001**, *130*, 17-20.
20. Jass, J.R.; Biden, K.G.; Cummings, M.C.; Simms, L.A.; Walsh, M.; Schoch, E.; Meltzer, S.J.; Wright, C.; Searle, J.; Young, J.; et al. Characterisation of a subtype of colorectal cancer combining features of the suppressor and mild mutator pathways. *J Clin Pathol* **1999**, *52*, 455-460.
21. Mirabelli-Primdahl, L.; Gryfe, R.; Kim, H.; Millar, A.; Luceri, C.; Dale, D.; Holowaty, E.; Bapat, B.; Gallinger, S.; Redston, M. Beta-catenin mutations are specific for colorectal carcinomas with microsatellite instability but occur in endometrial carcinomas irrespective of mutator pathway. *Cancer Res* **1999**, *59*, 3346-3351.
22. Dietmaier, W.; Wallinger, S.; Bocker, T.; Kullmann, F.; Fishel, R.; Ruschoff, J. Diagnostic microsatellite instability: definition and correlation with mismatch repair protein expression. *Cancer Res* **1997**, *57*, 4749-4756.
